# Granular cell tumor in the scrotum of a pediatric patient: A case report of a rare clinical entity^☆^

**DOI:** 10.1016/j.eucr.2023.102327

**Published:** 2023-02-07

**Authors:** Caleb Ashbrook, Shane F. Batie, Anita Sengupta, Craig A. Peters

**Affiliations:** a University of Texas Southwestern Medical Center, Department of Urology, 2001 Inwood Road, 4th Floor, West Campus Building, Dallas, TX, 75390, USA; b Children's Medical Center, Department of Urology, 2350 N Stemmons Freeway #F4300, Dallas, TX, 75207, USA; c Children's Medical Center, Department of Pathology, C1529, 1935 Medical District Drive, Dallas, TX, 75235, USA

**Keywords:** Granular cell tumor, Scrotum, Pediatrics

## Abstract

Granular cell tumors are rare tumors of Schwann cell origin that present in any anatomic location, age or sex. We present a case of a granular cell tumor in the scrotum of a prepubescent male. The tumor was excised, with histology revealing abundant eosinophilic cytoplasm and positive S-100 staining. No stigmata of malignancy were identified and no recurrence has been reported during follow-up.

## 1 Introduction

Granular cell tumors (GCTs) are rare tumors derived from Schwann cells that can be found in any anatomic location. Most commonly they are found in subcutaneous tissue and the gastrointestinal tract.^1^ They usually arise between the 4th and 6th decade in life and are more common in women.^1^^,^^2^ The presentation varies, but commonly occurs as a solitary, small painless nodule. The finding of multi-focal tumors has been associated with underlying syndromes (Noonan Syndrome, Neurofibromatosis I, and LEOPARD syndrome).^1^^,^^2^ While most tumors are benign, malignant lesions have been reported in 1–2% of cases, typically associated with high rates of metastasis and poor survival.^1^^,^^3^ Herein, we present a rare clinical entity of a GCT presenting as a solitary tumor in the scrotum of a pediatric patient.

## 2 Case presentation

A 7 year-old male was referred for a solitary scrotal nodule that had developed over the past year. There was no associated pain, drainage, antecedent trauma or prior history of surgery. He had no stigmata of underlying syndromic conditions.

**Fig. 1:**
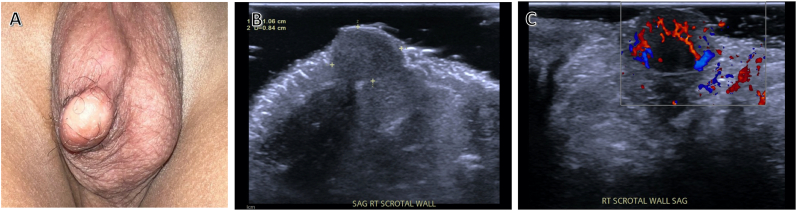
(A) Scrotal lesion on physical examination (B) Scrotal wall ultrasound demonstrating solid lesion (C) Color Doppler image showing internal vascular flow.. (For interpretation of the references to colour in this figure legend, the reader is referred to the Web version of this article.)

Physical examination revealed a normal, circumcised phallus with bilaterally descended testicles of normal size and consistency for his age. The lesion in question was a 1.5 × 1 cm superficial, epithelialized, firm mass just off midline in the mid-scrotum. The epithelium differed from the scrotal skin with a more pale, smooth surface, giving the lesion the appearance similar to a sebaceous cyst (Fig. 1A). An ultrasound revealed a solid superficial lesion with intrinsic flow (Fig. 1B; Fig. 1C).

Indications for excision were discussed with the family who elected to proceed. The patient underwent excision of the scrotal lesion, which was confined to the skin without deeper extension. An elliptical incision was made around the base of the lesion and incised sharply. Complete excision was carried out with blunt dissection and cautery. The skin defect was then closed in a multi-layer fashion.

**Fig. 2:**
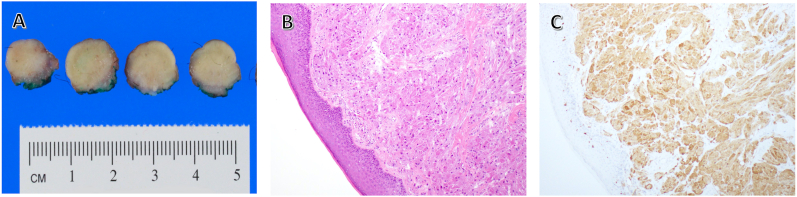
(A) Gross sections of lesion (post fixation) (B) H&E Staining at 10x magnification, demonstrating abundant eosinophilic cytoplasm and small nuclei (C) Positive staining for S-100, indicating neural origin of the lesion.

On gross examination, the lesion showed a firm white cut surface (Fig. 2A). The lesion was unencapsulated but circumscribed. Histologic examination revealed sheets and nests of large polygonal cells with abundant granular eosinophilic cytoplasm and small round nuclei (Fig. 2B). The cells were positive for S-100, indicative of neural origin (Fig. 2C).

## 3 Discussion

This case presentation highlights a rare clinical entity requiring a high index of suspicion to diagnose. Available literature regarding GCTs has largely come from small retrospective reviews, case series and case reports, though a recent systematic review by Mobarki et al. highlighted the variable clinical and histopathologic characteristics of these tumors.^4^

Granular cell tumors tend to present in women in the 4th to 6th decade in life but can occur at any age. Most present as solitary lesions, but approximately 12.5% are associated with trauma or chronic inflammation and 7.1% involve multiple lesions.^4^ Local recurrence after surgical resection occurs in 20.8% of cases with a positive surgical margin.^4^ Histopathologic findings vary significantly. The Fanburg-Smith criteria was published in 1998 with the goal of assessing clinical and histologic risk factors for malignancy and prognosis in GCTs, finding adverse prognostic features to be local recurrence, metastasis, large tumor size and older patient age. Histologic findings affecting prognosis included the presence of necrosis, high mitotic activity, tumor cell spindling, vesicular nuclei with large nucleoli, and >10% (corrected) Ki67 values.^3^ While narrowing of the criteria has been suggested, metastasis remains as the true indicator of malignant behavior. The identification of high risk features on pathology is key, as 1–2% of cases are malignant. Prognosis is poor for malignant GCTs, with 70% of patients at the time of the study having died from their disease and only 3.3% free of disease.^4^

Our patient had no clinical or histopathologic findings concerning for malignancy and is disease-free. GCT of the scrotum is rare, with few cases reported in the literature.^5^ Rarer still is the finding of a scrotal GCT in pediatric patients; to the best of our knowledge, there have been four other such reports. No pediatric scrotal GCT cases revealed a malignant tumor; however, malignant GCTs in the scrotum have been reported in adults (Table 1).

**Table 1:** Case reports of pediatric and malignant GCTs of the scrotum.

Patient Age (years)	Benign or Malignant	Solitary or Multifocal	Reference
15	Benign	Solitary	Richmond AM, La Rosa FG, Said S. Granular cell tumor presenting in the scrotum of a pediatric patient: a case report and review of the literature. *J Med Case Rep.* 2016; 10(1):161
12	Benign	Multi-focal	Alonso Calvar L, Plaza Alonso C, Alvarez Alvarez C, Ruger Jimenez L, Zarraonandia Andraca A, Ruibal Moldes M. [Scrotum-perineal granular cell tumor in pediatric age: A case report.]. *Arch Esp Urol.* 2021; 74(7):709-712
6	Benign	Solitary	Sidwell RU, Rouse P, Owen RA, Green JS. Granular cell tumor of the scrotum in a child with Noonan syndrome. *Pediatr Dermatol.* 2008; 25(3):341-343
10	Benign	Multi-focal	Oro-Ayude M, Mesa-Alvarez L, Alvarez-Alvarez C et al. Multifocal granular cell tumors in a 10-year-old boy. *Pediatr Dermatol.* 2021; 38(5):1374-1376
48	Malignant	Solitary	Behzatoglu K, Bahadir B. Malignant granular cell tumor with unusual histological features. *Pathol Int.* 2007; 57(2):115-119

## 4 Conclusion

Granular cell tumor of the scrotum in a child is rare, but clinical suspicion should be present in the evaluation of a genital lesion in pediatric males. Diagnosis of these tumors should prompt a thorough physical examination for multi-focal tumors, and if found, work-up for underlying genetic syndromes should be considered. Complete excision of the tumor should be considered given the malignant potential, especially in large tumors, followed by thorough histologic assessment geared towards the identification of risk factors for malignancy. In the absence of malignancy, the prognosis is very good with complete local excision.

## CRediT author statement

**Caleb Ashbrook**: Conceptualization, Writing – original draft **Shane F Batie**: Conceptualization, Writing – review/editing **Anita Sengupta**: Visualization, Writing – review/editing **Crag A Peters**: Supervision, Writing – review/editing.

## Declaration of competing interest

None.
